# ADGRL4/ELTD1 is a highly conserved angiogenesis-associated orphan adhesion GPCR that emerged with the first vertebrates and comprises 3 evolutionary variants

**DOI:** 10.1186/s12862-019-1445-9

**Published:** 2019-07-12

**Authors:** David M. Favara, Alison H. Banham, Adrian L. Harris

**Affiliations:** 1 Balliol College, University of Oxford, Oxford, UK; 2 Department of Oncology, University of Oxford, Oxford, UK; 3 Nuffield Division of Clinical Laboratory Sciences, Radcliffe Department of Medicine, University of Oxford, Oxford, UK

**Keywords:** ADGRL4, ELTD1, Adhesion GPCR evolution

## Abstract

**Background:**

Our laboratory identified ADGRL4/ELTD1, an orphan GPCR belonging to the adhesion GPCR (aGPCR) family, as a novel regulator of angiogenesis and a potential anti-cancer therapeutic target. Little is known about how ADGRL4/ELTD1 (and aGPCRs in general) function, a problem compounded by a lack of known ligands or means of activation. With this in mind, we turned to computational evolutionary biology with the aim of better understanding ADGRL4/ELTD1.

**Results:**

We identified *ADGRL4/ELTD1* as a highly conserved early angiogenic gene which emerged in the first true vertebrates (bony fish) approximately 435 million years ago (mya), evolving alongside key angiogenic genes *VEGFR2* and *DLL4*. We identified 3 evolutionary *ADGRL4/ELTD1* variants based on EGF domain deletions with variant 2 first emerging 101 mya (95% CI 96–105) in Afrotheria and 82 mya (95% CI 76–89) in Primates. Additionally, conservation mapping across all orthologues reveals highest level conservation in EGF Ca binding domain 1, suggesting that this motif plays an essential role, as well as specific regions of the GAIN domain, GPS motif and 7TM domain, suggesting possible activation mechanisms and ligand binding positions. Additionally, we found that *ADGRL4/ELTD1* (a member aGPCR family 1) is possibly ancestral to members of aGPCR family 2.

**Conclusion:**

This work establishes ADGRL4/ELTD1’s evolution, sheds light on its possible activation and ligand binding zones, and establishes the first temporal references for the emergence of ADGRL4/ELTD1 variants during vertebrate evolution. Our approach is applicable to the greater aGPCR family and opens up new avenues for future experimental work.

**Electronic supplementary material:**

The online version of this article (10.1186/s12862-019-1445-9) contains supplementary material, which is available to authorized users.

## Background

Discovered in 2001 [1], ELTD1/ADGRL4 is an orphan member of the G protein-coupled receptor (GPCR) superfamily, belonging to the 33 member ‘Adhesion GPCR’ family as per the Glutamate, Rhodopsin, Adhesion, Frizzled/Taste2, Secretin (GRAFS) classification [2]. Adhesion GPCRs (aGPCRs) are so named because unlike other GPCRs, they all contain large extracellular domains with adhesion motifs [3]. Human ELTD1/ADGRL4’s adhesion motifs comprise an epidermal growth factor (EGF) domain and an EGF Ca^2+^ binding domain. Recently, all aGPRCs underwent a nomenclature change [4] with ELTD1 being renamed ADGRL4. In this manuscript, all aGPCRS will be referred to by their new names followed by their old names (i.e. ADGRL4/ELTD1).

**Fig. 1:**
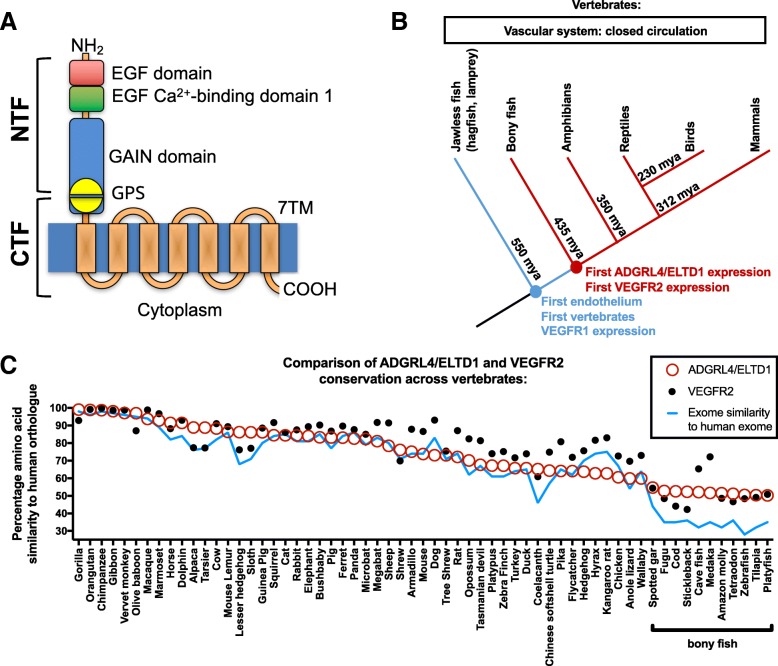
ADGRL4/ELTD1 is encoded in the genomes of bony fish and all subsequent vertebrates suggesting that it became established in vertebrates approximately 435 mya at the same time as VEGFR2. **a** Schematic representation of human ADGRL4/ELTD1’s putative structure showing NTF and CTF division. The NTF comprises an EGF-like domain and a Ca^2+^ binding EGF-like domain as well as the majority of the GAIN domain and N-terminal portion of the GPS. The CTF comprises the C-terminal portion of the GPS as well as the 7TM domain and a short intracellular ICD. **b** Cladogram depicting vertebrate evolution and the presence of orthologues to genomic *ADGRL4/ELTD1* and the core angiogenetic genes *VEGFR1* and *VEGFR2*. Blue represents the presence of orthologues to genomic V*EGFR1* without any *VEGFR2* or *ADGRL4/ELTD1*. Red indicates the presence of orthologues to genomic *VEGFR1*, *VEGFR2* and *ADGRL4/ELTD1*. Evolutionary divergence time estimates from [25, 26]. Cladogram not drawn to scale. **c** Comparison of ADGRL4/ELTD1 and VEGFR2 orthologue amino acid conservation across 61 vertebrate species with fully sequenced and annotated genomes reveals high ADGRL4/ELTD1 conservation across all vertebrates with both ADGRL4/ELTD1 and VEGFR2 following a similar trend. Each data point represents the percentage similarity of orthologue to human *ADGRL4/ELTD1* (red circle) or *VEGFR2* (black dot). The blue line (representing the percentage similarity of each species’ exome contrasted to the human exome) reveals that in bony fish, ADGRL4/ELTD1’s conservation is significantly higher than the exome conservation similarity suggesting that ADGRL4/ELTD1 is part of an early core angiogenic gene group and confirming previous in vivo experiments. (Abbreviations: GAIN = GPCR autoproteolysis inducing domain; GPS = GPCR proteolysis site)

Like other aGPCRS [5], ADGRL4/ELTD1’s predicted structure can be divided based on either topographical or cleavage-based compartmentation. Topographically, human ADGRL4/ELTD1 can be divided into three compartments: i) a large extra-cellular domain (ECD) containing an EGF domain, an EGF Ca binding domain (ADGRL4/ELTD1’s adhesion domains) and the ‘GPCR autoproteolysis inducing domain’ (GAIN) (which contains the GPCR proteolysis site (GPS)); ii) a 7-transmembrane domain (7TM); and iii) a short intracellular domain (ICD) [6]. Cleavage-based compartmentation centres on the GPS cleavage site and divides aGPCRs into an i) N-terminal Fragment (NTF) and ii) C-terminal fragment (CTF). A graphical representation is presented in Fig. 1a.

Our laboratories have previously identified ADGRL4/ELTD1 as a novel regulator of physiological and tumour angiogenesis [7]. This work revealed ADGRL4/ELTD1 to be upregulated in tumour-associated endothelial cells and vascular smooth muscle cells (head and neck, renal, colorectal and ovarian cancers) and to be essential for sprouting angiogenesis in both zebrafish and humans as well as being regulated by two key angiogenic ligands, namely upregulation by vascular endothelial growth factor (VEGF), and downregulation by the Notch ligand delta-like ligand 4 (DLL4). Additionally, *Adgrl4/Eltd1* silencing in ovarian and colorectal tumour xenografts in mice was found to substantially limit tumour growth by suppressing tumour-vessel angiogenesis. In human cancer patients, high tumour-vessel endothelial ADGRL4/ELTD1 expression was shown to correlate with improved overall survival (OS) in patients who subsequently received anti-cancer therapy (head and neck squamous carcinoma, renal, colorectal and ovarian cancers) implicating ADGRL4/ELTD1 as a putative prognostic marker of favourable outcome for these cancers types when treated with systemic therapy. This may be due to higher ADGRL4/ELTD1 expression correlating with higher microvessel density and vessel maturity, which thus allows for better systemic anti-cancer drug delivery to the tumour. ADGRL4/ELTD1 is not expressed by the majority of cancer cell lines [6], however it is highly upregulated in some glioblastoma tumour cells and has been shown to be important for glioblastoma cell survival, although the mechanism remains unclear [8–10].

Although *ADGRL4/ELTD1* is now recognised as an important angiogenesis gene in both zebrafish and humans, very little is known about ADGRL4/ELTD1’s function. This is further compounded by the general lack of knowledge surrounding other members of the aGPCR family as historically, the Adhesion family is the most poorly studied of the five GPCR families. This stems from the difficulty in activating these receptors, the majority of them being orphans that lack a known ligand. Recently, a growing number of orphan aGPCRs have had their activation mechanism solved using the Stachel Hypothesis [11–18]. This proposes that all expressed aGPCRs (except GPR123) contain a short 10–20 amino acid tethered agonist (the Stachel peptide) C-terminally to the GPS cleavage site which is essential for aGPCR activation through its action of striking the 7TM receptor loops and initiating signalling [11]. Despite this, ADGRL4/ELTD1’s activation remains unsolved.

With this in mind, we turned to computational evolutionary biology to try and better understand ADGRL4/ELTD1’s evolutionary history and identify domains with the highest conservation that are likely to be those involved in important biological functions. Previous work on the subject of GPCR evolution has revealed that the aGPCR group is ancient and is ancestral to the Secretin GPCR family, with the first Adhesion member arising in the amoeba *Dictyostelium discoideum* before diversifying in the free-living, basal metazoan *Trichoplax adhaerens* [19–21]. However, to date, no work has specifically investigated ADGRL4/ELTD1’s evolution.

The following aims underpin the rationale of our investigation:

1. To investigate *ADGRL4/ELTD1*’s evolution and conservation across extant sequenced species and to compare this to established angiogenesis core genes.
2. To investigate whether ADGRL4/ELTD1 has lost or gained any domains during its evolution (and if so, when?) and whether this is informative in regard to ADGRL4/ELTD1’s function.
3. To investigate and compare ADGRL4/ELTD1 to other closely related aGPCRS to shed light on shared ligands or additional functions as well as the origin of closely related families

## Results

### *ADGRL4/ELTD1* is a highly conserved early core angiogenic gene which arose approximately 435 million years ago within bony fish

The first question investigated was which is the oldest extant species whose genome encodes an *ADGRL4/ELTD1* orthologue? As ADGRL4/ELTD1 has been shown to be important in vessel sprouting, we hypothesised that the acquisition of *ADGRL4/ELTD1* would correlate with the development of endothelial cells in a closed circulatory system. The very first endothelial cells in a closed circulatory system arose approximately 550 million years ago (mya) with the first vertebrates: the jawless fish [22]. Today, members of this ancient group still exist: the hagfish and the lamprey. As the lamprey has been fully genome sequenced [23], it was chosen as the representative jawless fish and its annotated genome interrogated for the presence of an *ADGRL4/ELTD1* orthologue as well as orthologues of two important angiogenesis associated genes: *VEGFR1* (vascular endothelial growth factor receptor 1) and *VEGFR2* (vascular endothelial growth factor receptor 2). These two genes were chosen because of their core importance to the field of angiogenesis [24] and because ADGRL4/ELTD1 is known to be regulated by VEGF [7].

This analysis revealed that the lamprey encodes a precursor to *vegfr1* but not *adgrl4/eltd1* nor *vegfr2*. Following this, the next evolutionary vertebrate group (bony fish; *n* = 11 annotated genomes) as well as all evolutionarily subsequent vertebrate groups (total *n* = 61 annotated genomes) were interrogated for the presence of orthologues to *ADGRL4/ELTD1*, *VEGFR1* and *VEGR2*. Results revealed that *eltd1 and vegfr2* are present in the genomes of all bony fish and all subsequent vertebrates (Fig. 1b). The genomic presence of *VEGFR2* and *ADGRL4/ELTD1* orthologues in bony fish and all subsequent vertebrates suggests that these genes were acquired during the during the evolution of jawless fish to bony fish. In terms of evolutionary time, as bony fish appeared approximately 435 mya ago [25, 26], these results suggest that ADGRL4/ELTD1 and VEGFR2 became established in vertebrates during the same time period.

Next, ADGRL4/ELTD1’s conservation across vertebrate genomes was investigated. This was performed by comparing ADGRL4/ELTD1’s amino acid sequence to the longest predicted amino acid sequences of ADGRL4/ELTD1 orthologues in extant members of all the major vertebrate groups retrieved from Ensembl (*n* = 61 annotated genomes). As a comparison, this was also done for VEGFR2 and for the evolutionarily ancient angiogenesis-associated Notch signalling pathway ligand DLL4.

ADGRL4/ELTD1’s amino acid sequence is highly conserved across all 61 sequenced vertebrate genomes with even the least similar orthologues (bony fish) having a similarity of > 50% to human ADGRL4/ELTD1 (Fig. 1c). Additionally, ADGRL4/ELTD1’s conservation across all 61 species is similar to that of the highly conserved angiogenesis genes *VEGFR2* and *DLL4* (unpaired Student’s *t*-test *p* = 0.1 and *p* = 0.05). (Additional file 1: Figure S1). As a control, an extended panel of unrelated genes were reviewed, comprising 9 DNA repair genes and 16 receptor tyrosine kinases (RTK) (Additional file 1: Figure S1). Whilst the majority are as conserved as ADGRL4/ELTD1, some are significantly less conserved than ADGRL4/ELTD1, whilst a smaller minority are more conserved (Additional file 1: Figure S1A). This shows that the high-level conservation found in ADGRL4/ELTD1 and core angiogenesis regulators VEGFR2 and DLL4 is a common feature of many genes but does not apply uniformly to all genes. For the majority of species, exome similarity to the human exome follows a trend similar to ADGRL4/ELTD1 and VEGFR2. Interestingly, in all bony fish reviewed (*n* = 11 annotated genomes), *adgrl4/eltd1* is conserved to a far greater extent than the conservation of the fish exome (unpaired Student’s *t*-test *p* < 0.0001) with the same being true for the core angiogenic orthologues *vegfr2 and dll4* and the majority of the extended panel of important control genes (Additional file 1: Figure S1B).

### ADGRL4/ELTD1 has three evolutionary variants based on EGF domain deletions with 3 areas of major conservation indicating possible functional domains for future drug targeting

Next, the evolution of ADGRL4/ELTD1’s sequence during vertebrate evolution was investigated to shed light on conservation as a means to better understand ADGRL4/ELTD1’s domain function (e.g. which of ADGRL4/ELTD1’s domains are most critical for its function) in order to develop future cancer therapies targeting these domains. This was performed by aligning ADGRL4/ELTD1’s longest predicted amino acid sequences in 59 extant vertebrate species, representative of all major vertebrate groups from Bony Fish to Primates. An amino acid conservation score was then calculated for each aligned amino acid using a quantitative method which takes into account the physico-chemical properties of each aligned column [27]. Additionally, each sequence was scanned for conserved domains using the results from three amino acid sequence conservation databases and then annotated with domain information. Exon-intron data was retrieved from each orthologue’s genomic sequence and annotated with the domain information. Finally, results from the amino acid alignment were used to create a phylogenetic tree showing evolutionary distance between ADGRL4/ELTD1 orthologues.

**Fig. 2:**
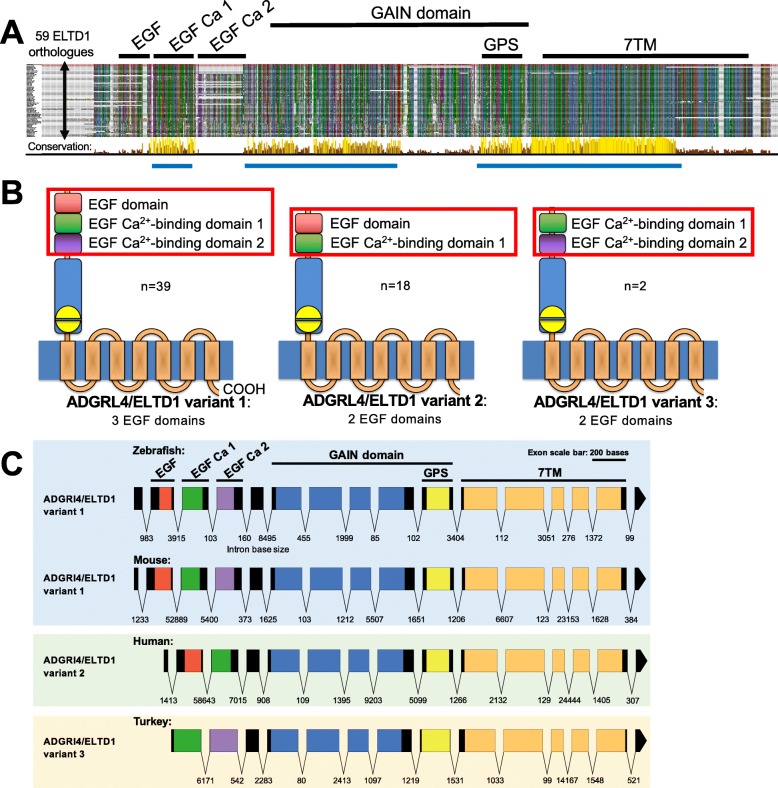
ADGRL4/ELTD1 comprises three evolutionary variants. **a** amino acid conservation across 59 extant vertebrate orthologues reveals 3 evolutionary ADGRL4/ELTD1 variants and three areas of major conservation: i) the EGF Ca binding domain; ii) GAIN domain and GPS motif; and iii) 7TM domain. Conservation scores are presented in the conservation track below the alignments; the highest scores being presented as yellow tall bars and the lowest with no bars. These results highlight ADGRL4/ELTD1’s 2 main evolutionary variants: the oldest (and majority) of ADGRL4/ELTD1 sequences comprise three EGF domains, whilst the more evolutionary recent species encode only 2 EGF domains. ADGRL4/ELTD1’s domains are annotated with black bars. Highly conserved areas are annotated with blue bars. Aligned genomes are listed starting with human (primate) continuing down the evolutionary ladder until platyfish (bony fish). **b** Graphical summary of ADGRL4/ELTD1’s three evolutionary variants showing the frequency of each variant in the sample of orthologues examined. **c** Exon map of representative vertebrates encoding ADGRL4/ELTD1 variants 1–3 showing that each EGF and EGF Ca binding domain segregates to its own exon. In vertebrates which only express ADGRL4/ELTD1 variant 2, during evolution the fourth exon was likely deleted from ADGRL4/ELTD1 variant 1 producing ADGRL4/ELTD1 variant 2. In the case of ADGRL4/ELTD1 variant 3, the first two exons were removed. Exons are drawn to scale whilst intron sizes are listed between each exon and are not drawn to scale. Domains are listed and are colour coded

Results, presented as a colour coded sequence alignment, reveal high-level conservation across all analysed ADGRL4/ELTD1 orthologues (Fig. 2a). In this sequence alignment, ADGRL4/ELTD1 orthologues are stacked in evolutionary order, with Primates at the top and bony fish at the bottom. Strikingly, the most heavily conserved areas were i) the first EGF Ca^2+^ binding domain, ii) the N-terminal half of the GAIN domain which includes the GPS motif, and iii) the majority of the 7TM domain (Fig. 2b). ADGRL4/ELTD1 predicted splice variants were then reviewed in all species revealing that ADGRL4/ELTD1 appears to have three evolutionary variants based on EGF domain deletions. This revealed that the ADGRL4/ELTD1 sequence containing the most domains (termed ADGRL4/ELTD1 variant 1) was present in all predicted bony fish species splice variants, but not in all subsequent evolutionary groups. We used this finding as the foundation for our model to interrogate ADGRL4/ELTD1 domain evolution in younger species finding that ADGRL4/ELTD1’s EGF domains are deleted in more recent species. The oldest, as well as the majority of vertebrate ADGRL4/ELTD1 orthologues encode three EGF domains (termed ADGRL4/ELTD1 variant 1) comprising: i) EGF domain (most N-terminal EGF domain); ii) EGF Ca^2+^ binding domain 1; and iii) EGF Ca^2+^ binding domain 2 (Fig. 2b). The majority of Primates (including humans) and other more recent vertebrates are missing EGF Ca^2+^ binding domain 2 and only encode two EGF domains (termed ADGRL4/ELTD1 variant 2) comprising: i) EGF domain; and ii) EGF C^a2+^ binding domain 1 (Fig. 2b). A minority of ADGRL4/ELTD1 orthologues (*n* = 8) were predicted to express transcripts of both *ADGRL4/ELTD1* Variant 1 and Variant 2 (Additional file 1: Table S1). Two vertebrates (zebra finch and platypus) exhibited changes suggestive of a third ADGRL4/ELTD1 variant (termed ADGRL4/ELTD1 variant 3), which did not contain the first EGF Ca^2+^ binding domain, whilst containing both EGF Ca^2+^ binding domain 1 and 2 (Fig. 2b). Only one variant (duck) was predicted to express transcripts of all three ADGRL4/ELTD1 variants (Additional file 1: Table S1). Analysis of each ADGRL4/ELTD1 orthologue’s exon map revealed that each EGF and EGF Ca^2+^ binding domain segregate to their own exon. In receptors which only express ADGRL4/ELTD1 variant 2, this suggests that during vertebrate evolution, ADGRL4/ELTD1 variant 1’s 4th exon was deleted producing variant 2 (Fig. 2c). In the case of ADGRL4/ELTD1 variant 3, the first two exons were deleted (Fig. 2c). Interestingly, all orthologues maintain the EGF Ca^2+^ binding domain 1 which is the only EGF domain which is unaffected by deletion, signifying its probable importance in receptor activation and function.

A snake-plot depicting human ADGRL4/ELTD1’s domains with the highest conservation across all orthologues is presented in Additional file 1: Figure S2. These comprise the EGF Ca^2+^ domain 1, the N-terminal half of the GAIN domain, the majority of the GPS motif, and the first five transmembrane loops of the 7TM. As canonical GPCR signalling occurs when a ligand binds to the extracellular loops of the 7TM, it can be hypothesised that ADGRL4/ELTD1’s 7TM extracellular loops with the highest evolutionary conservation (loops 2–3 and 4–5) could potentially the most important for ligand binding leading to signal transduction. As ADGRL4/ELTD1’s activation and signalling remains unsolved, the above external 7TM loops present prime opportunities for using mutagenesis or antibody targeting experiments to determine how ADGRL4/ELTD1 is activated. A phylogenetic tree showing the amino acid evolutionary distance between the above ADGRL4/ELTD1 orthologues reveals that each ADGRL4/ELTD1 orthologue localises to a grouping representing their respective evolutionary vertebrate group (Additional file 1: Figure S2). Furthermore, the phylogenetic tree shows how evolutionary similar mammalian ADGRL4/ELTD1 sequences are to each other, despite differences in EGF domain number in ADGRL4/ELTD1 variant 1 and 2 forms of the receptor. All predicted amino acid domain mapping details for each orthologue are listed in Additional file 1: Table S1.

### ADGRL4/ELTD1 variant 2 first appeared approximately 101 million years ago within Afrotheria and then within Primates 82 million years ago

Next, we investigated when ADGRL4/ELTD1’s variants could have developed during vertebrate evolution. To answer this, an evolutionary phylogenetic timetree (a phylogenetic tree superimposed with robust estimates of evolutionary time) was created for the 59 extant vertebrate species analysed above. A minimum of two vertebrates from each major group were included. Evolutionary time estimates were derived from the curated “Timetree: timescales of life” database [26] with annotations added to this detailing which ADGRL4/ELTD1 variant is encoded in each species. The model for determining the approximate initial time period for the emergence of ADGRL4/ELTD1 variants 2 and 3 relied on the assumption that ADGRL4/ELTD1 variant 1 was the first form of ADGRL4/ELTD1. Evolutionary time was then measured from the earliest point of divergence from an ancestor common to an ADGRL4/ELTD1 variant 1 and variant 2 or 3 expressing vertebrate (in other words, from the point of emergence of an ancestor unique to an ADGRL4/ELTD1 variant 2 or 3 encoding vertebrate).

**Fig. 3:**
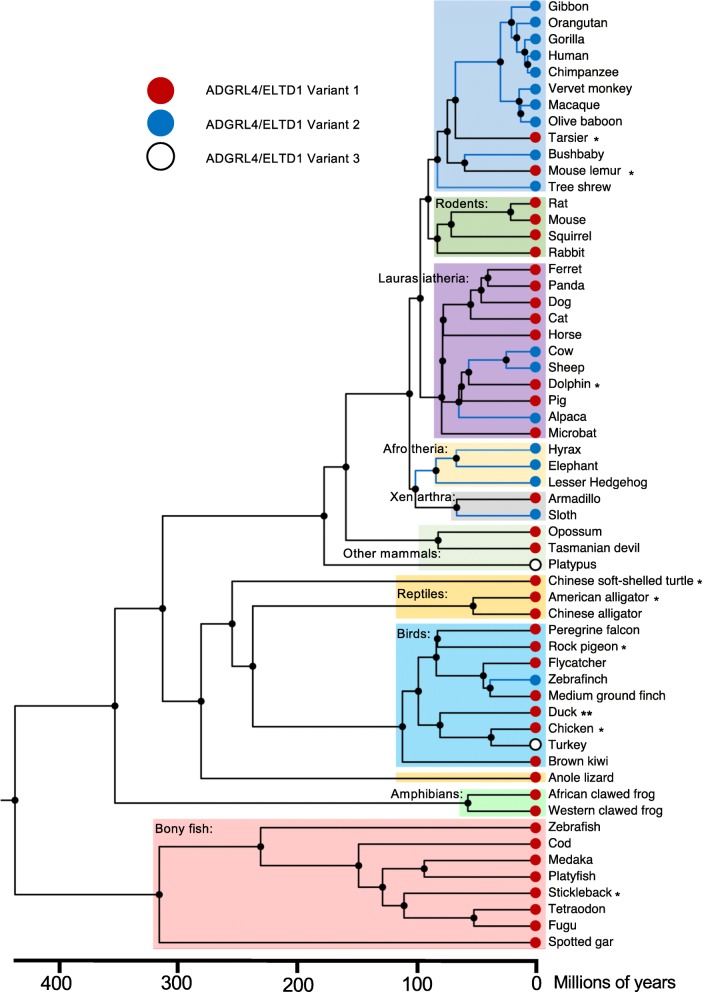
Evolutionary phylogenetic timetree representing evolution in extant vertebrate species showing the emergence of ADGRL4/ELTD1 variants. Red terminal nodes indicate ADGRL4/ELTD1 variant 1; blue terminal nodes indicate ADGRL4/ELTD1 variant 2; and the white circles indicate the low frequency ADGRL4/ELTD1 variant 3. Blue phylogenetic lines indicate the emergence of ADGRL4/ELTD1 variant 2 expression according to the model. The earliest vertebrates to encode ADGRL4/ELTD1 variant 2 were the three included mammals from the Afrotheria group (hyrax, elephant and lesser hedgehog) diverging from their common ADGRL4/ELTD1 variant 1 encoding ancestor approximately 101 million years ago. * indicates animals which express both ADGRL4/ELTD1 variant 1 and 2 according to NCBI RefSeq or Ensembl. ** indicates animals which expresses all three ADGRL4/ELTD1 variants (only duck in this data set)

This analysis (Fig. 3) revealed that the earliest extant true vertebrates (bony fish) all are predicted to express ADGRL4/ELTD1 variant 1, with only 1 member predicted to express both ADGRL4/ELTD1 variant 1 and 2. The only publically available fully sequenced cartilaginous fish is the elephant shark. As our model for vertebrate ADGRL4/ELTD1 variant evolution required a minimum of two organisms per vertebrate class, we have not included the elephant shark in our evolutionary timetree. Interestingly, the elephant shark is predicted to express ADGRL4/ELTD1 variant 2. Sequencing of additional members of this group will determine whether this is representative for all cartilaginous fish. The amphibian group only contained ADGRL4/ELTD1 variant 2 although the sample size is smaller. The very earliest vertebrates to encode only ADGRL4/ELTD1 variant 2 were the three included mammals from the Afrotheria group (hyrax, elephant and lesser hedgehog) who diverged from their common ADGRL4/ELTD1 variant 1 encoding ancestor in Africa approximately 101 mya (95% CI 96–105) during the Cretaceous period (145–65 mya). The first ADGRL4/ELTD1 variant 2 encoding Primate diverged from their ADGRL4/ELTD1 variant 1 encoding common ancestor approximately 82 mya (95% CI 76–89) with the majority of the Primate group encoding ADGRL4/ELTD1 variant 2 (*n* = 10/12). Next, approximately 66 mya (95% CI 60–72), the sloth diverged from its ADGRL4/ELTD1 variant 1 encoding common ancestor. The ADGRL4/ELTD1 variant 2 expressing members of the Laurasiatheria group diverged from their common ADGRL4/ELTD1 variant 1 encoding ancestor approximately 62 mya (95% CI 58–66) (during the Paleogene Period (65–23 mya) (Fig. 3). Within birds, ADGRL4/ELTD1 variant 2 has only more recently started to be expressed by 1 member: the zebra finch, whose ancestor diverged from ADGRL4/ELTD1 variant 1 encoding descendants approximately 38 mya (95% CI 16–43) (Paleogene Period). ADGRL4/ELTD1 variant 3 is encoded in the platypus, which evolved from its common ADGRL4/ELTD1 variant 1 encoding ancestor approximately 177 mya (95% CI 163–191) during the Jurassic Period (199–145 mya) and in the turkey, which evolved from its common ADGRL4/ELTD1 variant 1 encoding ancestor approximately 37 mya (95% CI 28–39) (Paleogene Period). Whether these represent a true third ADGRL4/ELTD1 variant remains unclear because of their low frequency amongst sampled vertebrates. Of all investigated vertebrates, only the duck was predicted to express all 3 ADGRL4/ELTD1 variants.

### ADGRL4/ELTD1 is a hybrid comprising elements common to both aGPCR family 1 and 2 and is possibly ancestral to family 2 aGPCRs

**Fig. 4:**
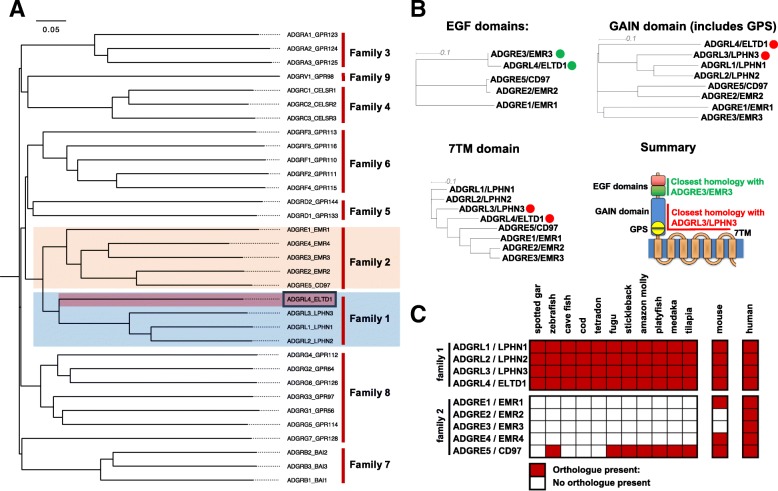
ADGRL4/ELTD1 is most similar to ADGRL3/LPHN3 and ADGRE3/EMR3 and is likely to be an evolutionary ancestral to members of aGPCR family 2. **a** Phylogenetic tree displaying the genetic difference between all aGPCRS showing that aGPCR family 1 (which contains ADGRL4/ELTD1) shares its common ancestor with family 2. **b** Phylogenetic comparisons between domains belonging to members of aGPCR Family 1 and Family 2 reveals that ADGRL4/ELTD1’s EGF domains are most closely related to ADGRE3/EMR3’s EGF domains and ADGRL4/ELTD1’s GAIN domain, GPS motif and 7TM domain are most similar to the respective domains in ADGRL3/LPHN3. The coloured dots highlight ADGRL4/ELTD1 and the closest relative for the domain being compared amongst all compared members. The colouring represents whether the closest relative (for the domain being compared) is a member of aGPCR family 1 (red) or 2 (green). **c** Heatmap detailing the presence of orthologue in the first true vertebrates (bony fish) reveals that ADGRL4/ELTD1 evolved before the incorporation of aGPCR family 2 into the vertebrate genome and because of its domain composition is likely to be ancestral to members of aGPCR family 2

Human ADGRL4/ELTD1 was compared to other members of the human aGPCR family in order to shed light on potentially shared ligands or functions and to understand the origin of closely related GPCR families. This was performed by contrasting the amino acid sequences of all aGPCRS and plotting phylograms. Thereafter, domain specific phylogenetic comparisons were performed. Phylogenetic comparison of the amino acid sequences of all human aGPCRs separates all members into their respective families (this was the basis for the original categorisation into distinct families performed by [2]). This shows that aGPCR family 1 (of which ADGRL4/ELTD1 is a member) shares its common ancestor with aGPCR family 2 (Fig. 4a). Phylogenetic comparison of the amino acids of all murine aGPCRs reveals the same result (Additional file 1: Figure S3A). All members of aGPCR family 2 contain EGF domains on their N-terminal fragment (NTF), as does ADGRL4/ELTD1. Interestingly, ADGRL4/ELTD1’s EGF adhesion motifs are not found in the other aGPCR family 1 members (ADGRL1–3/LPHN1–3). Reciprocally, the rhamnose-binding lectin (RBL), olfactomedin (OLF) and hormone receptor motif (HRM) domains found in ADGRL1–3/LPHN1–3 are not found on ADGRL4/ELTD1.

We then compared the similarity of ADGRL4/ELTD1’s predicted domains to those expressed on the members of aGPCR family 1 and 2. Results reveal that ADGRL4/ELTD1’s EGF domains are most similar to those of aGPCR family 2’s ADGRE3/EMR3 receptor (Fig. 4b). As members of aGPCR family 4 also contain EGF domains (in addition to cadherin repeats and laminin G domains), a comparison of all aGPCR EGF domains (ADGRL4/ELTD1, family 2 members and family 4 members) was conducted with the same result (Additional file 1: Figure S3B). In addition, ADGRL4/ELTD1’s GAIN domain, GPS motif and 7TM domain are most similar to aGPCR family 1’s ADGRL3/LPHN3 receptor (Fig. 4b).

These results, combined with the knowledge that the majority of aGPCR families segregate into groups containing extracellular domains unique to that family, suggest that ADGRL4/ELTD1 is a hybrid comprising domain elements from both aGPCR family 1 and 2. As regards possible shared ligands, although ligands to ADGRE3/EMR3 remain unknown, the EGF motif ligands of other family 2 members are known, namely: dermatan sulfate [28] for both ADGRE2/EMR2 and ADGRE5/CD97; and CD55 [29], CD90 [30], and integrins α5β1, avβ3 [31] for ADGRE5/CD97. Interestingly, none of these molecules has been shown to activate signalling in these receptors and, although none are known to associate with ADGRL4/ELTD1, they present a way forward for initiating ADGRL4/ELTD1’s ligand experiments.

The above ADGRL4/ELTD1 domain homology data beg the question: which developed first, the members of aGPCR family 1 or those of GPCR family 2 and could ADGRL4/ELTD1 possibly be ancestral to members of family 2? This question was approached by searching for orthologues of both aGPCR family 1 and family 2 members in early vertebrates. Results reveal that the lamprey genome contains a precursor to a single member of aGPCR family 1 (a precursor to ADGRL2/LPHN2 or ADGRL3/LPHN3) but none to any member of aGPCR family 2 (Fig. 4c). All bony fish genomes reviewed (*n* = 11) contained orthologues to all members of aGPCR family 1. In contrast, only 1 member of aGPCR family 2 was found as an orthologue in these genomes (ADGRE5/CD97). Looking forward in evolutionary time, the mouse similarly encodes all family 1 members with a larger repertoire of family 2 members, whilst the human genome contains all members of both families (Fig. 4c). These results suggest that Family 1 is likely to be the older of the two families, and that ADGRL4/ELTD1 was likely present before the first members of Family 2 evolved, suggesting the possibility of ADGRL4/ELTD1 being ancestral to members of aGPCR family 2 through having shared some of its EGF exons with the evolving members of family 2. The close similarity between ADGRL4/ELTD1’s EGF domains with ADGRE3/EMR3’s EGFs suggests that ADGRE3/EMR3 is the most recent descendant of ADGRL4/ELTD1.

## Discussion

Since the advent of the human genome project, several studies have been undertaken to determine the aGPCR repertoire in vertebrates using the following species as model organisms: human, rat, mouse, chicken, zebrafish and pufferfish [3, 32–37]. These studies clearly showed that not all vertebrates contained homologs to all the human aGPCR repertoire and that *ADGRL4/ELTD1* was present in all 6 reviewed organisms. Whilst contributing greatly to the understanding and classification of aGPCRs, these were limited by the number of available sequenced vertebrates at the time. ADGRL4/ELTD1 was first discovered in rat and human cardiomyocytes, with rat being noted to have 1 EGF domain and 2 EGF Ca binding domains whilst human contained 1 EGF domain and 1 EGF Ca binding domain [1]. Since this initial discovery, no further systematic work has been undertaken to explore ADGRL4/ELTD1’s variants with the aim of understanding ADGRL4/ELTD1’s function. Studies performed in a range of ancient invertebrates have revealed that aGPCRs are of ancient origin. In ancient invertebrates however, many aGPCRS are species-specific, comprising a smaller limited range of human aGPCR homologs which do not include ADGRL4/ELTD1 [19, 20, 38–43].

The current study builds on this previous evolutionary aGPCR work. Firstly, it systematically interrogates ADGRL4/ELTD1’s homologues within a far wider and more representative range of vertebrates as well as confirming that ADGRL4/ELTD1 first emerged with the evolution of the first true vertebrates. Secondly, this study systematically reviews ADGRL4/ELTD1’s sequence and domain evolution across a wide range of vertebrate homologs, establishing a temporal reference for the emergence of ADGRL4/ELTD1 variants during vertebrate evolution, and investigating whether ADGRL4/ELTD1 is ancestral to other aGPCRS. The study provides novel insights into ADGRL4/ELTD1’s evolution, its variants, potential functional elements as well as means of activation, and its relationship to other aGPCRs. These insights provide many useful ideas not only for future ADGRL4/ELTD1 studies but also for investigating the remainder of the aGPCR family, which remain poorly functionally understood due to the majority having orphan status with no known ligand. This work reveals that ADGRL4/ELTD1 arose within the first true vertebrates and has remained present in the genomes of all subsequent vertebrates. Additionally, ADGRL4/ELTD1 appears to be as highly conserved as members of the VEGF and Notch angiogenic pathways, key pathways which our laboratory has previously shown to affect ADGRL4/ELTD1 expression [7]. Interestingly, it can be hypothesised that the minority of vital DNA repair and RTK genes which are significantly less conserved than ADGRL4/ELTD1 have evolved more complex interactions and are therefore less conserved, whilst those that are more conserved play a more fundamental early role in cellular biology. Similar to other core angiogenic genes, ADGRL4/ELTD1’s higher orthologue-human similarity compared to orthologue-human exome similarity in bony fish suggests that *adgrl4/eltd1* has an important biological role in fish. This is supported by the finding that *adgrl4/eltd1* silencing in zebrafish embryos is lethal, causing major disruption to the developing vasculature (although this data was obtained with morpholinos, we used two antisense morpholinos targeting the start codon and a splicing junction of the *adgrl4/eltd1* pre-mRNA) [7]. The finding that *Adgrl4/Eltd1* knockout in murine embryos is not lethal or disruptive to the vasculature either as a single gene knockout [44] or as a double knockout in combination with the adhesion GPCR Adgrf5/Gpr116 [45] suggests that higher vertebrate genomes have incorporated additional protective redundancy against such events (i.e. the incorporation of additional angiogenic genes). Taken together, this positions ADGRL4/ELTD1 as an evolutionarily early angiogenic gene in vertebrates. It remains an open question whether ADGRL4/ELTD1 and the above-mentioned angiogenesis genes reciprocally affected each other’s evolution. To investigate this, future experiments are needed to interrogate whether ADGRL4/ELTD1 EGF domain losses within individual species are linked to any structural changes in VEGF or Notch genes or any intermediaries – or vice versa. The hypothesis that ADGRL4/ELTD1 possibly arose in the first endothelial cells is based on ADGRL4/ELTD1 being expressed nearly exclusively by endothelial cells [7] and that both endothelial cells [22] and ADGRL4/ELTD1 emerged in the first vertebrates.

The finding that ADGRL4/ELTD1 across vertebrates comprises three evolutionary variants with a minority of vertebrates expressing more than 1 ADGRL4/ELTD1 variant is also novel and sheds light on ADGRL4/ELTD1’s important domains and possible means of activation. As more data becomes available, this opens the door to future RNASeq studies investigating the expression of these variants across a wide range of vertebrates. Interestingly, in all vertebrates, ADGRL4/ELTD1’s EGF Ca^2+^ binding domain 1 is never deleted. This high-level conservation suggests that this is possibly the most important ‘adhesion’ domain of ADGRL4/ELTD1’s multiple EGF adhesion domains and is most likely to be involved in receptor activation and function. Although ADGRL4/ELTD1’s ligand remains unknown, we hypothesise that it most likely interacts with an EGF domain. The finding that more recent vertebrates have lost an EGF domain suggests that this probable ligand is able to sufficiently activate the receptor through its interaction with only one or two EGF domains. It is also probable that the ligand(s) itself has evolved and changed conformation, prompting a change in the number of EGFs required for activation.

The recently proposed ‘Stachel’ Hypothesis states that each aGPCR contains a tethered agonist located C-terminal to the GPS point of cleavage (located at the N-terminal end of the CTF) (Fig. 1a) [11]. Furthermore, the GAIN domain including its GPS motif are important for the function of the GPS cleavage site [46]. Although ADGRL4/ELTD1’s activation and signalling mechanism(s) remain unknown, a growing number of aGPCRS have been shown to function through the Stachel mechanism [11–15, 18]. In these examples, aGPCR signalling is initiated when the tethered agonist (or a ligand analogous to that amino acid sequence) binds to a specific extracellular portion of the 7TM (the exact binding region remaining unsolved at present). ADGRL4/ELTD1’s high level GAIN domain and GPS motif conservation across all vertebrates suggests that ADGRL4/ELTD1‘s activation might follow a similar mechanism. With this in mind, one can use ADGRL4/ELTD1’s conservation mapping across orthologues to hypothesise that its highly conserved external 7TM regions (external loops 2–3, and 4–5) could potentially represent functionally important sites for ligand binding and the possible initiation of ADGRL4/ELTD1’s signal transduction (which remains unsolved). Once ADGRL4/ELTD1’s signalling is better understood, this finding presents ample opportunity for future experiments including 7TM mutation or antibody activating/blocking experiments targeting these specific 7TM regions in order to reveal exactly which residues are important for agonist/antagonist binding. Additionally, conservation mapping across vertebrate orthologues can also be used to guide receptor activation studies in other aGPCRs.

The evolutionary timetree findings are the first temporal approximations of ADGRL4/ELTD1’s evolution within vertebrates. These findings present an interesting view into the past and strengthens the suggestion that ADGRL4/ELTD1’s EGF domain and EGF Ca^2+^ binding domain 2 are evolutionarily expendable and that losing them has been evolutionarily beneficial for newer species as no ADGRL4/ELTD1 orthologue regains these domains once a common ancestor has lost them. As the EGF Ca binding domain 1 is present in every examined *ADGRL4/ELTD1* orthologue, it can be hypothesised that this is most functionally important of ADGRL4/ELTD1’s EGF domains and should be targeted in future ADGRL4/ELTD1 functional experiments. It is also worth noting that there have been no other domain deletions or major additions apart from the EGF domain to ADGRL4/ELTD1’s sequence during vertebrate evolution. Exactly why the number of EGF domains has decreased as vertebrates have evolved remains open to speculation. A possible hypothesis includes the consideration that this has occurred in response to an evolutionary modification of ADGRL4/ELTD1’s yet unsolved ligand(s) or through evolutionary pruning of excess or redundant domains. A minority of vertebrates were predicted to express more than one ADGRL4/ELTD1 variant, suggesting that these receptors are in the midst of evolutionary change. Interestingly, only two vertebrate genomes in our dataset were predicted to express ADGRL4/ELTD1 variant 3 only: the platypus (which diverged from its common ancestor 177 mya and the zebrafinch, which diverged from its common ancestor most recently 38 mya. Why this has happened and whether these represent a true third ADGRL4/ELTD1 variant remains unclear because of their low frequency amongst our sampled vertebrates. Although it appears that ADGRL4/ELTD1 variants 2 and 3 have been selected across multiple vertebrate groups, it is possible that a different picture will appear when a larger repertoire of vertebrate genomes is available for assessment in the future.

This study also sheds light on the possible origins of aGPCR family 2, suggesting that ADGRL4/ELTD1’s exons containing EGF domains are possibly ancestral to this family. Although aGPCR family 2 members are mostly expressed by immune cells, there is a known functional overlap between ADGRL4/ELTD1 and at least one family 2 member: AGRE5/CD97, which stimulates angiogenesis in an integrin-dependant manner [31]. The close EGF similarity between ADGRL4/ELTD1 and members of aGPCR family 2 suggests the possibility that the extracellular matrix (ECM) ligands known to bind to members of aGPCR family 2 may also bind to ADGRL4/ELTD1, opening the door to future functional experiments. This possibility is further strengthened by the ECM itself being known to play a key role in regulating angiogenesis [47]. A limitation to this hypothesis is that EGF domains are found in many proteins not sharing homology to ADGRL4/ELTD1 and that family 2’s EGFs may have been acquired from a different ancestor. The close similarity between ADGRL4/ELTD1’s EGFs and those in family 2 make this less likely.

## Conclusion

This work reveals that the orphan aGPCR receptor ADGRL4/ELTD1 is a highly conserved early angiogenic gene which emerged in the vertebrate genome during the evolution of bony fish approximately 435 mya with a similar high-level conservation to established core regulators of angiogenesis. This work further establishes the first temporal references for the emergence of three ADGRL4/ELTD1 variants during vertebrate evolution, based on EGF domain deletions. These ADGRL4/ELTD1 variant findings establish the importance of EGF Ca binding domain 1 as the adhesion domain most likely to be important for ADGRL4/ELTD1’s functional activity. Results from conservation mapping describe the most likely external 7TM sites important for ligand binding and subsequent receptor activation, which present useful target sites for future in vitro angiogenesis activation/blocking experiments. Results show that ADGRL4/ELTD1 contains structural elements common to both aGPCR family 1 and 2 and that ADGRL4/ELTD1 may possibly be ancestral to members of family 2. In summary, these findings open up new horizons and ideas for further experiments to better understand both ADGRL4/ELTD1, and other members of the historically poorly studied aGPCR family.

## Methods

ADGRL4/ELTD1’s evolutionary bioinformatics work was performed using the Ensembl database of publically available annotated, sequenced genomes [48] as well as with BioMart [49]. All nucleotide coding sequences and protein sequences as well as similarity comparisons between human ADGRL4/ELTD1, VEGFR2, DLL4 and BRCA1 and its orthologues were extracted from Ensembl. Amino acid sequences were extracted from NCBI RefSeq and Ensembl. Orthologues were extracted from Ensembl (which uses gene order conservation scoring as well as whole genome alignment scoring to assign orthologues). Exome comparisons were also extracted from Ensembl (which uses pairwise whole-genome comparisons to make comparisons between the human genome and other genomes). Furthermore, Ensembl was used to interrogate splice variants. Each sequence was scanned for conserved domains using three amino acid sequence conservation databases (Pfam using KEGG motif [50], SMART [51] and CDD [52]). For comparison of two groups, the Student’s *t*-test was used. For unpaired data, an unpaired Student’s *t*-test was performed. For comparison of two or more groups, a one-way ANOVA was computed. Significance is denoted as: * *p* ≤ 0.05, ** *p* ≤ 0.01, *** *p* ≤ 0.001, **** *p* ≤ 0.0001. All sequence alignment was performed using Clustal Omega [53] using either nucleotide or amino acid sequences as stated. Conservation scores were calculated using Jalview [54]. Sequence conservation scanning was performed as mentioned above. Phylograms and cladograms were computed from the Clustal outputs and arranged using Dendroscope [55]. Evolutionary timetree data was extracted from the public Timetree.org database [26]. Orthologues and homologues of all interrogated genes in extant organisms were extracted from the Ensembl and KEGG databases [50].

## Additional file

Additional file 1: **Figure S1 A)** Percentage similarity to human orthologue across all 61 orthologues reveals that ADGRL4/ELTD1 is as highly conserved as VEGFR2 and DLL4 as well as the majority of highly evolutionarily important DNA damage repair and receptor tyrosine kinase genes, whilst a minority are less conserved than ADGRL4/ELTD1 and a smaller minority are more conserved. Comparisons with ADGRL4/ELTD1 using unpaired Student’s *t*-test, significance denoted as: * *p* ≤ 0.05, ** *p* ≤ 0.01, *** *p* ≤ 0.001, **** *p* ≤ 0.0001. **B)** Percentage similarity to human orthologue across 11 fish genomes reveals that *adgrl4/eltd1* is conserved to a far greater extent than the conservation of the fish exome with the same being true for core angiogenic genes and the majority of unrelated evolutionarily important DNA repair and receptor tyrosine kinase genes. Comparisons to exome similarity, using unpaired Student’s *t*-test, significance denoted as: * *p* ≤ 0.05, ** *p* ≤ 0.01, *** *p* ≤ 0.001, **** *p* ≤ 0.0001. Abbreviations: ns = non-significant. **Figure S2 A)** Human ADGRL4/ELTD1 amino acid residue diagram highlighting areas of high conservation across ELTD1 orthologues. Domains with the highest conservation comprise the EGF Ca^2+^ domain, the N-terminal half of the GAIN domain, the majority of the GPS motif, and the first five transmembrane loops of the 7TM, the external portion of which possible ligand binding or receptor activation occurs. ADGRL4/ELTD1’s signal peptide sequence as well as all domains are colour coded and are detailed in the legend. **B)** Phylogenetic tree depicting amino acid similarity and evolutionary distance between ADGRL4/ELTD1 orthologues in 59 vertebrate species; schematic drawn to scale. **Figure S3 A)** Phylogenetic tree displaying the genetic difference between all murine aGPCRS showing that aGPCR family 1 (which contains ADGRL4/ELTD1) shares its common ancestor with family 2. **B)** Phylogenetic comparisons between EGF domains belonging to members of aGPCR Family 1, Family 2 and Family 5 reveals that ADGRL4/ELTD1’s EGF domains are most closely related to ADGRE3/EMR3’s EGF domains. **Table S1.** ADGRL4/ELTD1 orthologues and their predicted protein sequences, extracted from NCBI RefSeq and Ensembl databases. Colour coding relates to ADGRL4/ELTD1 variant expression: Red shading = ADGRL4/ELTD1 variant 1; Blue shading = ADGRL4/ELTD1 variant 2; Grey shading = ADGRL4/ELTD1 variant 3; Green shading = species expressing splice variants of both ADGRL4/ELTD1 variant 1 and 2. (DOCX 1110 kb)

## Data Availability

The datasets used and/or analysed during the current study are available from the corresponding author on reasonable request.

## Abbreviations

7TM: 7-transmembrane domain
aGPCRs: Adhesion GPCRs
CTF: C-terminal fragment
DLL4: Delta-like ligand 4
EGF: Epidermal growth factor
GAIN: GPCR autoproteolysis inducing domain
GPS: GPCR proteolysis site
GRAFS: Glutamate, Rhodopsin, Adhesion, Frizzled/Taste2, Secretin
ICD: Intracellular domain
NTF: N-terminal Fragment
VEGF: Vascular endothelial growth factor
