# Supplementary material for: ADGRL4/ELTD1 is a highly conserved angiogenesis-associated orphan adhesion GPCR that emerged with the first vertebrates and comprises 3 evolutionary variants
Source: BMC Evol Biol. 2019 Jul 12;19:143. doi: 10.1186/s12862-019-1445-9 (PMC6626334; doi:10.1186/s12862-019-1445-9)
Supplement: Supplementary file 1 — Figure S1 A) Percentage similarity to human orthologue across all 61 orthologues reveals that ADGRL4/ELTD1 is as highly conserved as VEGFR2 and DLL4 as well as the majority of highly evolutionarily important DNA damage repair and receptor tyrosine kinase genes, whilst a minority are less conserved than ADGRL4/ELTD1 and a smaller minority are more conserved. Comparisons with ADGRL4/ELTD1 using unpaired Student’s t-test, significance denoted as: * p ≤ 0.05, ** p ≤ 0.01, *** p ≤ 0.001, **** p ≤ 0.0001. B) Percentage similarity to human orthologue across 11 fish genomes reveals that adgrl4/eltd1 is conserved to a far greater extent than the conservation of the fish exome with the same being true for core angiogenic genes and the majority of unrelated evolutionarily important DNA repair and receptor tyrosine kinase genes. Comparisons to exome similarity, using unpaired Student’s t-test, significance denoted as: * p ≤ 0.05, ** p ≤ 0.01, *** p ≤ 0.001, **** p ≤ 0.0001. Abbreviations: ns = non-significant. Figure S2 A) Human ADGRL4/ELTD1 amino acid residue diagram highlighting areas of high conservation across ELTD1 orthologues. Domains with the highest conservation comprise the EGF Ca2+ domain, the N-terminal half of the GAIN domain, the majority of the GPS motif, and the first five transmembrane loops of the 7TM, the external portion of which possible ligand binding or receptor activation occurs. ADGRL4/ELTD1’s signal peptide sequence as well as all domains are colour coded and are detailed in the legend. B) Phylogenetic tree depicting amino acid similarity and evolutionary distance between ADGRL4/ELTD1 orthologues in 59 vertebrate species; schematic drawn to scale. Figure S3 A) Phylogenetic tree displaying the genetic difference between all murine aGPCRS showing that aGPCR family 1 (which contains ADGRL4/ELTD1) shares its common ancestor with family 2. B) Phylogenetic comparisons between EGF domains belonging to members of aGPCR Family 1, Family 2 and Family 5 reveals th [file 12862_2019_1445_MOESM1_ESM.docx]

## Supplementary material

**Supplementary Figure S1**: **A)** Percentage similarity to human orthologue across all 61 orthologues reveals that ADGRL4/ELTD1 is as highly conserved as VEGFR2 and DLL4 as well as the majority of highly evolutionarily important DNA damage repair and receptor tyrosine kinase genes, whilst a minority are less conserved than ADGRL4/ELTD1 and a smaller minority are more conserved. Comparisons with ADGRL4/ELTD1 using unpaired Student's *t*-test, significance denoted as: * *p* ≤ 0.05, ** *p* ≤ 0.01, *** *p* ≤ 0.001, **** *p* ≤ 0.0001. **B)** Percentage similarity to human orthologue across 11 fish genomes reveals that *adgrl4/eltd1* is conserved to a far greater extent than the conservation of the fish exome with the same being true for core angiogenic genes and the majority of unrelated evolutionarily important DNA repair and receptor tyrosine kinase genes. Comparisons to exome similarity, using unpaired Student's *t*-test, significance denoted as: * *p* ≤ 0.05, ** *p* ≤ 0.01, *** *p* ≤ 0.001, **** *p* ≤ 0.0001. Abbreviations: ns = non-significant.

**Supplementary figure S2:** **A)** Human ADGRL4/ELTD1 amino acid residue diagram highlighting areas of high conservation across ELTD1 orthologues. Domains with the highest conservation comprise the EGF Ca^2+^ domain, the N-terminal half of the GAIN domain, the majority of the GPS motif, and the first five transmembrane loops of the 7TM, the external portion of which possible ligand binding or receptor activation occurs. ADGRL4/ELTD1’s signal peptide sequence as well as all domains are colour coded and are detailed in the legend. **B)** Phylogenetic tree depicting amino acid similarity and evolutionary distance between ADGRL4/ELTD1 orthologues in 59 vertebrate species; schematic drawn to scale.

**Supplementary figure S3: A)** Phylogenetic tree displaying the genetic difference between all murine aGPCRS showing that aGPCR family 1 (which contains ADGRL4/ELTD1) shares its common ancestor with family 2. **B)** Phylogenetic comparisons between EGF domains belonging to members of aGPCR Family 1, Family 2 and Family 5 reveals that ADGRL4/ELTD1’s EGF domains are most closely related to ADGRE3/EMR3’s EGF domains

**Supplementary Table S1:** ADGRL4/ELTD1 orthologues and their predicted protein sequences, extracted from NCBI RefSeq and Ensembl databases. Colour coding relates to ADGRL4/ELTD1 variant expression: Red shading = ADGRL4/ELTD1 variant 1; Blue shading = ADGRL4/ELTD1 variant 2; Grey shading = ADGRL4/ELTD1 variant 3; Green shading = species expressing splice variants of both ADGRL4/ELTD1 variant 1 and 2.
